# Re-evaluating basophil count as a hematological indicator for bone density: a subgroup analysis from an East Asian population

**DOI:** 10.3389/fendo.2025.1643760

**Published:** 2025-09-04

**Authors:** Yu-Li Wang, Shu-Wei Huang, Jun-Jie Hong, Tiffany Wang, Kuei-Chen Lee, Chao-Min Cheng

**Affiliations:** ^1^ Department of Surgery, Hualien Armed Force General Hospital, Hualien, Taiwan; ^2^ International Intercollegiate Ph.D. Program, National Tsing Hua University, Hsinchu, Taiwan; ^3^ Department of Applied Science, National Taitung University, Taitung, Taiwan; ^4^ Inti Taiwan, Inc., Hsinchu, Taiwan; ^5^ Department of Physical Medicine and Rehabilitation, Tri-Service General Hospital, National Defense Medical University, Taipei, Taiwan; ^6^ Institute of Biomedical Engineering, National Tsing Hua University, Hsinchu, Taiwan

**Keywords:** basophil count, bone mineral density (BMD), osteoporosis screening, alanine aminotransferase (GPT), hematologic biomarker, metabolic bone disease, East Asian population, low-cost screening tool

## Abstract

**Background:**

While traditional risk factors for osteoporosis such as age, sex, and menopause are well-established, emerging evidence suggests that immune cells may also influence bone metabolism. Among them, the role of basophils remains poorly understood. This study investigated the association between peripheral blood basophil count and lumbar spine bone mineral density (BMD) in an East Asian adult population.

**Methods:**

A retrospective analysis was conducted on 200 adults undergoing health check-ups and lumbar dual-energy X-ray absorptiometry. Basophil count and other hematologic and biochemical parameters were correlated with lumbar spine T-scores using multivariate regression.

**Results:**

Basophil count showed no significant correlation with T-scores in the overall cohort (r = 0.06, p = 0.4261). However, a weak inverse trend was noted in participants with BMI ≥ 27. In contrast, GPT and creatinine were significantly associated with BMD, with alanine aminotransferase (GPT) emerging as a strong independent predictor (β = 0.61, p < 0.001).

**Conclusions:**

Basophil count does not appear to be a reliable biomarker for BMD in the general population. However, findings in the higher-BMI subgroup suggest a potential link that warrants further investigation. GPT may hold greater utility as a surrogate marker for bone health in clinical screening. The present findings also highlight the value of publishing negative results and underscore the need for future research in larger and more diverse cohorts.

## Introduction

Lower back disease is a prevalent condition that affects individuals across all demographics, irrespective of age, gender, or occupation (1–4). Its etiology is multifactorial, encompassing both spinal and non-spinal origins. Among spinal causes, lumbar spine disorders—such as intervertebral disc herniation, spondylolisthesis, and degenerative changes—are particularly common, especially among the elderly and athletes. Osteoporosis, a systemic skeletal disorder characterized by low bone mass and microarchitectural deterioration, represents a major global public health concern, currently affecting over 200 million individuals and accounting for approximately 8.9 million fracture cases annually (5). These musculoskeletal conditions not only cause chronic pain and reduced quality of life but are frequently associated with depression and may lead to severe comorbidities (6, 7).

In recent years, growing attention has been directed toward the study of musculoskeletal system degeneration, which is believed to compromise spinal stability and impair functional balance. Despite extensive investigation, the pathogenesis of osteoporosis remains incompletely elucidated and warrants further exploration. Numerous studies have demonstrated significant associations between reduced bone mineral density (BMD) and variables such as age, gender, and serum biochemical parameters, all of which may influence vertebral integrity and lumbar spine biomechanics (8–12). A comprehensive summary of clinically recognized serum biochemical markers and electrolyte/hormonal parameters associated with osteoporosis is provided in **Table 1** (13–18). These markers are frequently utilized in the diagnostic evaluation of metabolic bone diseases and serve as supportive indicators for identifying secondary causes of reduced BMD, assessing fracture risk, and monitoring treatment response.

**Table 1:** Biochemical markers and electrolytes associated with osteoporosis.

Marker	Reference range (Adults)	Clinical relevance in osteoporosis
Total Calcium	8.5–10.5 mg/dL (2.12–2.62 mmol/L)	Hypocalcemia stimulates PTH secretion, enhancing osteoclastic activity and bone resorption. Hypercalcemia may indicate primary hyperparathyroidism.
Ionized Calcium	4.4–5.3 mg/dL (1.1–1.3 mmol/L)	Reflects physiologically active calcium levels; more accurate than total calcium in altered pH states.
Phosphate	2.5–4.5 mg/dL (0.81–1.45 mmol/L)	Hyperphosphatemia suppresses active vitamin D synthesis and contributes to bone loss, especially in chronic kidney disease.
Magnesium	1.7–2.4 mg/dL (0.70–0.99 mmol/L)	Hypomagnesemia impairs PTH secretion and action, leading to functional hypoparathyroidism and bone loss.
Parathyroid Hormone (PTH)	10–65 pg/mL	Elevated PTH levels suggest primary or secondary hyperparathyroidism, a common cause of bone turnover and osteoporosis.
25-Hydroxy Vitamin D	30–50 ng/mL (optimal) >20 ng/mL (sufficient)	Vitamin D deficiency impairs calcium absorption and is a key risk factor for decreased bone mineral density.

This table summarizes common serum biochemical and hormonal markers associated with bone metabolism and osteoporosis, including minerals, electrolytes, and regulatory hormones. These parameters are frequently used in clinical evaluations for secondary causes of low bone mineral density or for monitoring metabolic bone diseases (13–18).

The pathophysiology of lumbar spine disorders is inherently complex, involving an interplay of age-related degeneration, genetic predisposition, and chronic mechanical stress on the lumbar vertebrae. Moreover, increasing evidence implicates inflammatory processes in the development of osteoporosis (19–21). The emerging field of osteoimmunology, introduced in 2000, has garnered significant interest for its potential to elucidate the interplay between immune regulation and bone remodeling (21–25). Osteoimmunology has emerged as a promising field revealing the complex interplay between immune cells and bone metabolism. Recent studies have proposed peripheral blood markers such as monocyte and basophil counts, as well as erythrocyte sedimentation rate (ESR), as potential predictors of chronic low back pain and advanced disc degeneration (26–32). Inflammatory activity is now widely acknowledged to contribute to bone loss, with neutrophil-to-lymphocyte ratio (NLR) being one of the most consistently reported systemic inflammatory markers negatively correlated with BMD. Elevated NLR has been particularly associated with increased osteoporosis risk in postmenopausal women (33–38).

Additionally, emerging evidence suggests that allergic and immunologic responses may modulate bone metabolism. Basophils, though traditionally associated with allergic responses, are now recognized as key contributors to chronic inflammation through the release of cytokines such as IL-4 and IL-13. This prolonged inflammatory state may have indirect effects on bone remodeling, although the precise mechanisms remain to be clarified (39–41).

These mediators are thought to influence osteoclast differentiation and activity, thereby promoting bone resorption and contributing to bone mass reduction. Although IL-4 and IL-13 are known to exert anti-osteoclastogenic effects under certain conditions, basophil-derived histamine and leukotrienes may conversely enhance bone resorption (42).

Therefore, the net impact of basophils on bone metabolism may depend on the inflammatory context and metabolic status (27, 30, 43, 44). Recent studies have highlighted the interplay between systemic inflammation and bone health. For instance, the systemic immune-inflammation index (SII) has been associated with decreased bone mineral density and increased risk of osteoporosis, particularly in postmenopausal women. Moreover, inflammatory markers such as interleukin-6 and C-reactive protein (CRP) have been correlated with bone density and strength (45–47). While NLR and SII are well-established inflammation-related markers for osteoporosis, we were unable to compute these indices in our cohort due to incomplete lymphocyte data. Despite these plausible biological mechanisms, the role of basophils in bone metabolism remains underexplored in current literature, especially in East Asian populations (28, 48–50). Given these findings, the present study aims to examine whether the association between peripheral basophil count and BMD is modulated by BMI in an East Asian cohort, and to explore potential interactions with metabolic parameters. Therefore, the present study focuses on investigating the association between peripheral basophil concentration and BMD, particularly T-scores, in an East Asian cohort (50, 51). Through this approach, we aim to shed light on potential immunologic contributions to osteoporosis pathogenesis and identify novel prognostic markers or therapeutic targets in this demographic. While prior studies focused on selected populations, such as postmenopausal women, this study examines a general East Asian cohort to explore broader immunometabolic associations with bone mineral density, including potential modifiers like BMI and metabolic markers.

In addition to understanding immune mechanisms, the clinical utility of identifying simple, readily available blood biomarkers for bone health assessment has attracted increasing attention (52). Although DEXA remains the gold standard for BMD assessment, it is not universally available and is often constrained by health insurance coverage. There is, therefore, growing interest in integrating routine hematological parameters (e.g., GPT, creatinine, and basophil count) into early screening algorithms or prediction models (46, 52–54). Studies exploring these biomarkers may aid in the development of low-cost, population-wide risk stratification tools, especially in resource-limited settings.

## Materials and methods

### Study design and case collection

This retrospective observational study was conducted to evaluate the association between BMD and laboratory serological parameters in adult patients at an eastern regional teaching hospital in Taiwan. The protocol was approved by the Institutional Review Board of Tri-Service General Hospital (IRB No. C202405032), and all procedures complied with the Declaration of Helsinki. Patient data were anonymized prior to analysis to ensure confidentiality.

Clinical information was extracted from the hospital’s Picture Archiving and Communication System (PACS) and electronic medical records. Patients who underwent dual-energy X-ray absorptiometry (DEXA) (HOLOGIC ASY-05119, USA (Normal HVL@140kVp with added filtration: 14.0 mm AI(Discovery Wi/Ci) Normal added filtration @ 140kVp 6.8mm AL equiv. (Discovery Wi/Ci)) for lumbar spine BMD between January 1 and December 30, 2024, were screened. Of 402 initially identified cases, 200 patients with complete clinical and laboratory data were included in the final analysis.

### Inclusion and exclusion criteria

The inclusion criteria were adults aged ≥18 years who received lumbar spine BMD evaluation using DEXA. Exclusion criteria included:

1. Age <18 years.
2. History of anti-osteoporotic medication use.
3. History of spinal vertebroplasty or cement augmentation.
4. Incomplete or missing laboratory data.

### Variables and data processing

Primary outcomes included lumbar spine T-score and Z-score (patient BMD - average BMD for age, gender, and ethnicity)/standard deviation. Laboratory parameters analyzed comprised complete blood count (Sysmex Automated Hematology Analyzer, Type: XN1500, Japan) (including basophil, neutrophil, and white blood cell counts), hepatic function markers (aspartate aminotransferase (GOT), alanine aminotransferase (GPT)), renal function markers (BUN, creatinine, estimated glomerular filtration rate (eGFR)), and fasting glucose. Baseline demographics (age, gender, height, weight, BMI) were also included.

Data preprocessing involved handling missing values (n = 36) using the k-nearest neighbors (kNN) imputation algorithm (55) to preserve both sample size and the distribution of the variables. Outliers (n = 93) were identified using the interquartile range (IQR) method, with values below Q1 − 1.5 × IQR or above Q3 + 1.5 × IQR considered extreme and replaced with the respective upper or lower bounds. All continuous variables were log_2_-transformed prior to modeling to improve normality and stabilize variance.

### Statistical analysis

Descriptive statistics were computed for all demographic, hematologic, and biochemical variables. Outliers were identified and adjusted using IQR method, while missing values were imputed via KNN algorithm to retain sample size and distribution integrity. Continuous variables were log_2_-transformed when appropriate to normalize distributions and improve model performance.

Pearson correlation coefficients were calculated to evaluate linear associations between BMD and individual variables, with the lumbar spine T-score serving as the primary outcome. One-way analysis of variance (ANOVA) was used to compare variables across World Health Organization (WHO)-defined BMD categories: normal (T ≥ −1.0); osteopenia (−2.5 < T < −1.0); and, osteoporosis (T ≤ −2.5). Variables included in these group-based comparisons were age, height, weight, BMI, creatinine, GPT, BUN, GOT, eGFR, and selected hematologic indices (e.g., WBC, basophils). Although BMI, height, BUN, GOT, and eGFR showed significant trends in univariate or ANOVA analyses, they were excluded from multivariate regression models due to high multicollinearity with other predictors.

Multivariate linear regression models were constructed to identify independent predictors of lumbar T-score. Covariates included basic physical characteristics, hematological parameters, and biochemical markers. The final model was selected based on conceptual relevance and statistical criteria. Multicollinearity among independent variables was evaluated using Pearson correlation matrices, and variables with strong intercorrelations were removed to ensure model stability. Standardized beta coefficients were reported.

To address imbalanced subgroup sizes, stratified random sampling based on T-score categories (T ≤ −2.0, −2.0 < T < 0, and T ≥ 0) was employed to construct training and test sets for model validation. Subgroup analyses stratified by sex and BMI (≥ 27 vs. < 27) were also conducted to explore potential effect modification. Principal component analysis (PCA) was applied to evaluate data clustering and detect outlier patterns based on log-transformed biochemical variables.

Statistical significance was defined as a two-sided p-value < 0.05. All analyses were performed using R (v4.2.3) or equivalent statistical software platforms.

## Results

**Figure 1:**
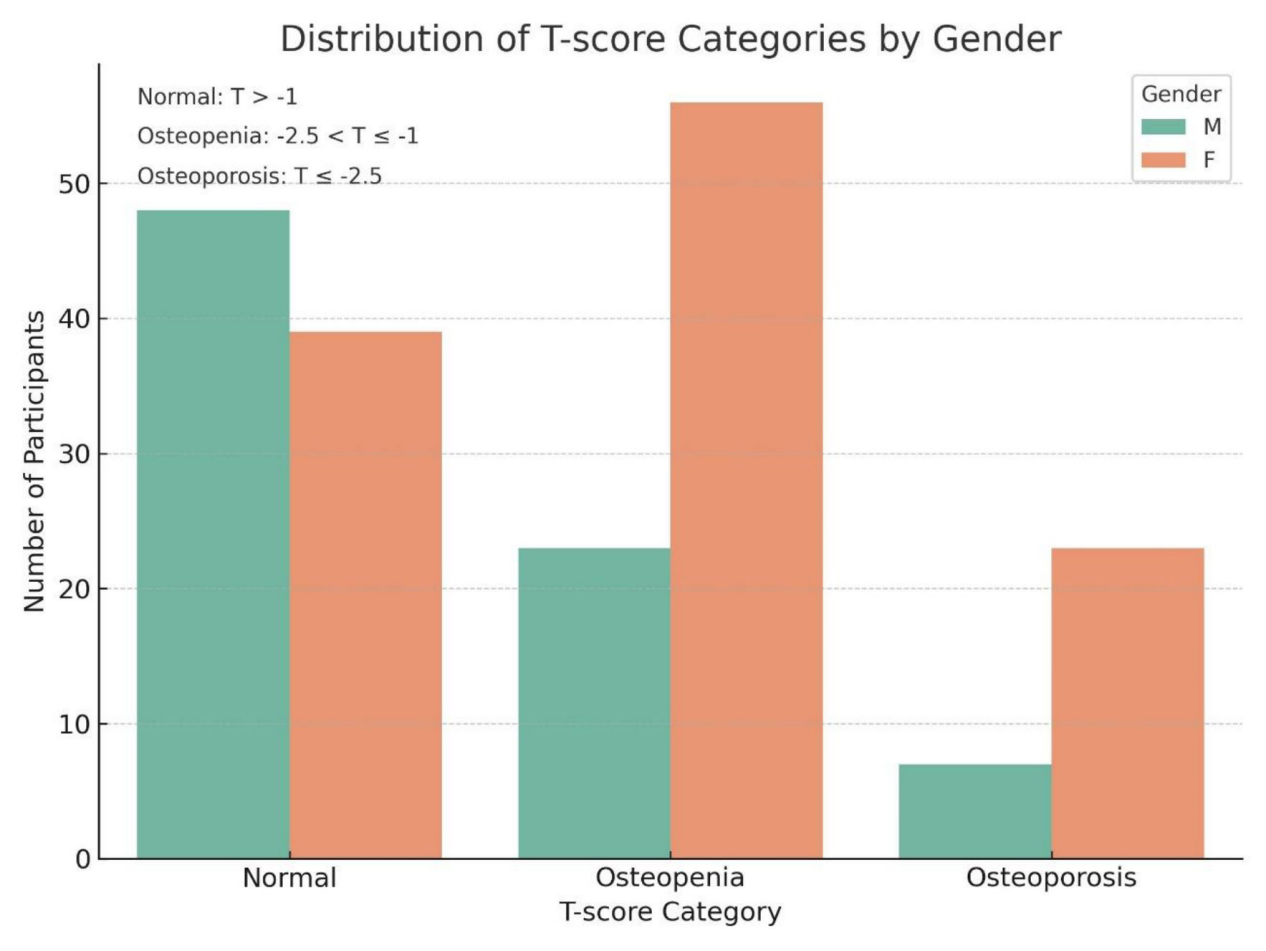
The bar chart illustrates the distribution of participants across three bone mineral density (BMD) categories-normal (T-score > -1), osteopenia (- 2.5 <T-score <-1), and osteoporosis (T-score <-2.5)-stratified by gender. A higher proportion of males exhibited normal BMD, while females were more frequently represented in the osteopenia and osteoporotic groups. This pattern highlights a greater burden of low bone mass among female participants, consistent with known sex-based differences in osteoporosis risk.

After exclusion of incomplete cases from the initial cohort (N = 402), a total of 200 eligible adult participants were included in the final analysis (male: female = 79: 121) (**Table 2**). Based on the WHO criteria for BMD, participants were stratified into three diagnostic categories: normal (T ≥ −1.0), osteopenia (−2.5 < T < −1.0), and osteoporosis (T ≤ −2.5). The gender-based distribution of T-score categories is illustrated in **Figure 1**, which reveals a higher prevalence of osteopenia and osteoporosis among female participants.

**Table 2:** Characteristics of the study participants.

Variable	Value
Gender (Male: Female)	79: 121
Overall Age (years)	60.9 ± 12.5¹
Male Age	58.6 ± 13.1¹
Female Age	62.4 ± 12.1¹
Overall T-score	-1.11 ± 1.36¹
Male Group T-score	-0.51 ± 1.32¹
Female Group T-score	-1.48 ± 1.25¹

¹Mean ± standard deviation (SD).

This table summarizes the baseline demographic and bone mineral density (BMD) characteristics of the 200 participants included in the final analysis. The cohort consisted of 79 males and 121 females. Mean age and T-score values are reported overall and stratified by sex. Female participants exhibited a lower mean T-score compared to males, consistent with the higher prevalence of low bone mass and osteoporosis reported in women.

**Figure 2:**
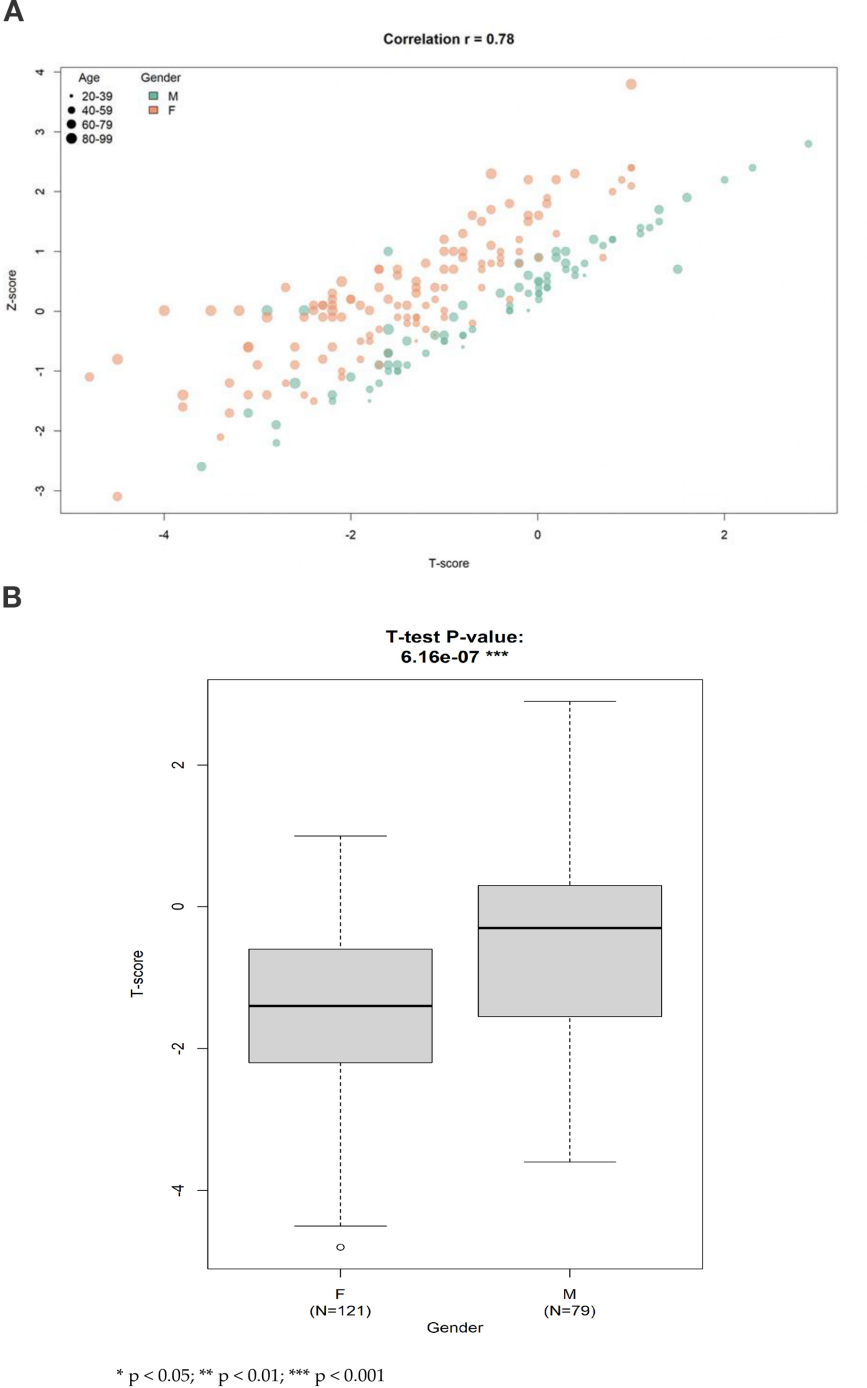
**(A)** The scatter plot illustrating the linear correlation between T-score and Z-score in the study population. A strong positive relationship was observed (Pearson's correlation coefficient r = 0.78), indicating that both indices consistently reflect lumbar spine bone mineral status. This supports the internal validity of bone density measurement across different standardization methods within the cohort. **(B)** Multivariate regression summary highlighting standardized beta coefficients and 95% confidence intervals for variables associated with lumbar spine T-score. Age and female sex were significantly associated with lower T- scores. GPT remained a significant positive predictor after adjustment, while basophil count was not statistically significant. These results emphasize the stronger predictive value of hepatic function and demographic factors compared to inflammatory markers.

A strong positive linear correlation was observed between T-score and Z-score (Pearson’s r = 0.78), indicating high internal consistency between BMD indices. Female participants exhibited significantly lower mean T-scores compared to their male counterparts (p = 6.2e−7) (**Figure 2**).

**Figure 3:**
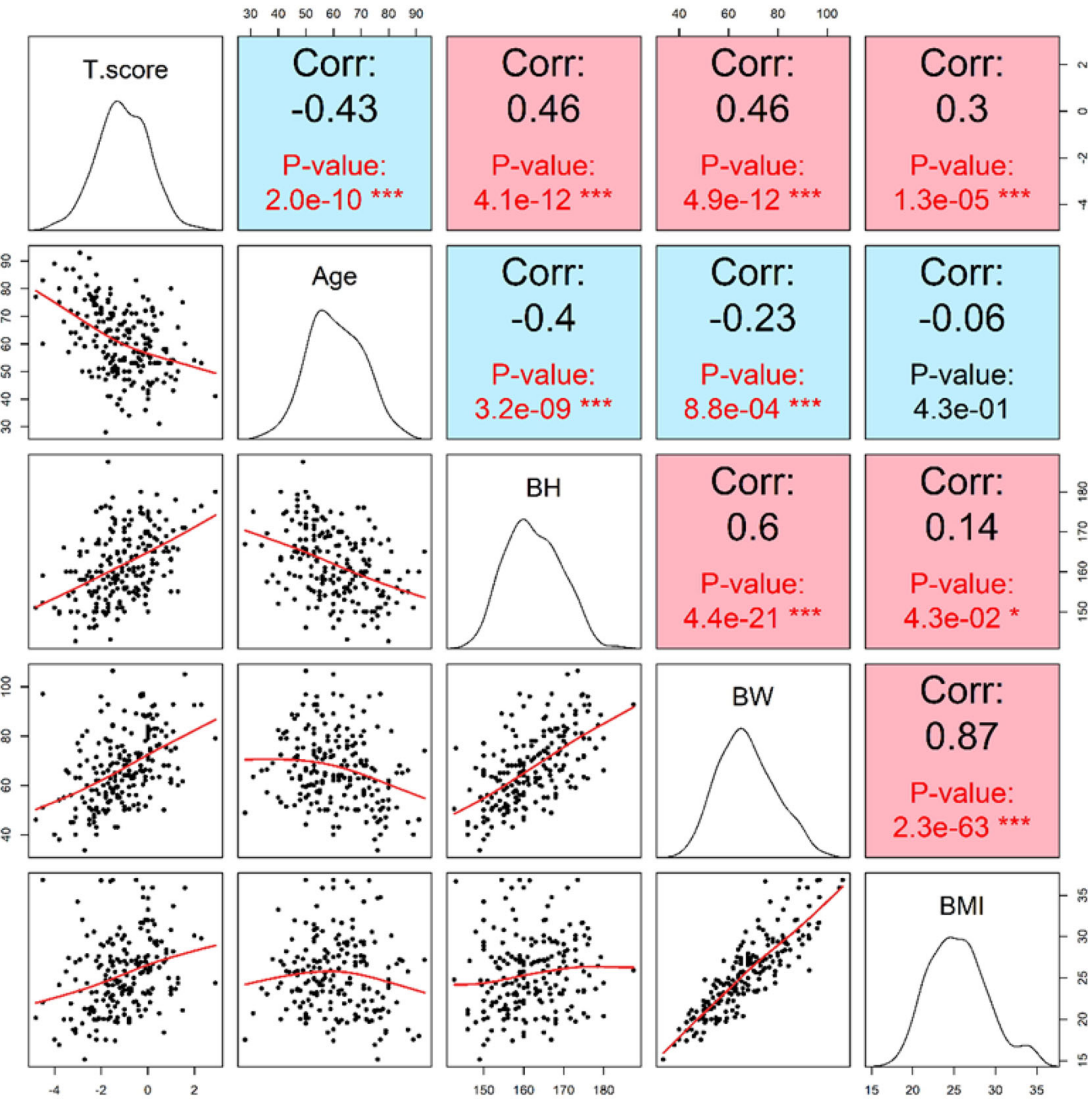
Scatterplots illustrating significant univariate correlations between lumbar spine T-score and basic anthropometric variables. Significant correlations were observed between lumbar spine T-score and age, body height (BH), body weight (BW), and body mass index (BMI). Age demonstrated a moderate negative correlation with T-score (r = -0.43, p = 2.0e-10), indicating an age-related decline in bone mineral density. In contrast, both BH and BW showed moderate positive correlations (r = 0.46, p = 4.1e-12 and r = 0.46, p = 4.9e-12, respectively), while BMI showed a weaker but still significant positive correlation (r=0.30, p = 1.3e-5). These findings emphasize the influence of age and body composition on bone health.

**Figure 4:**
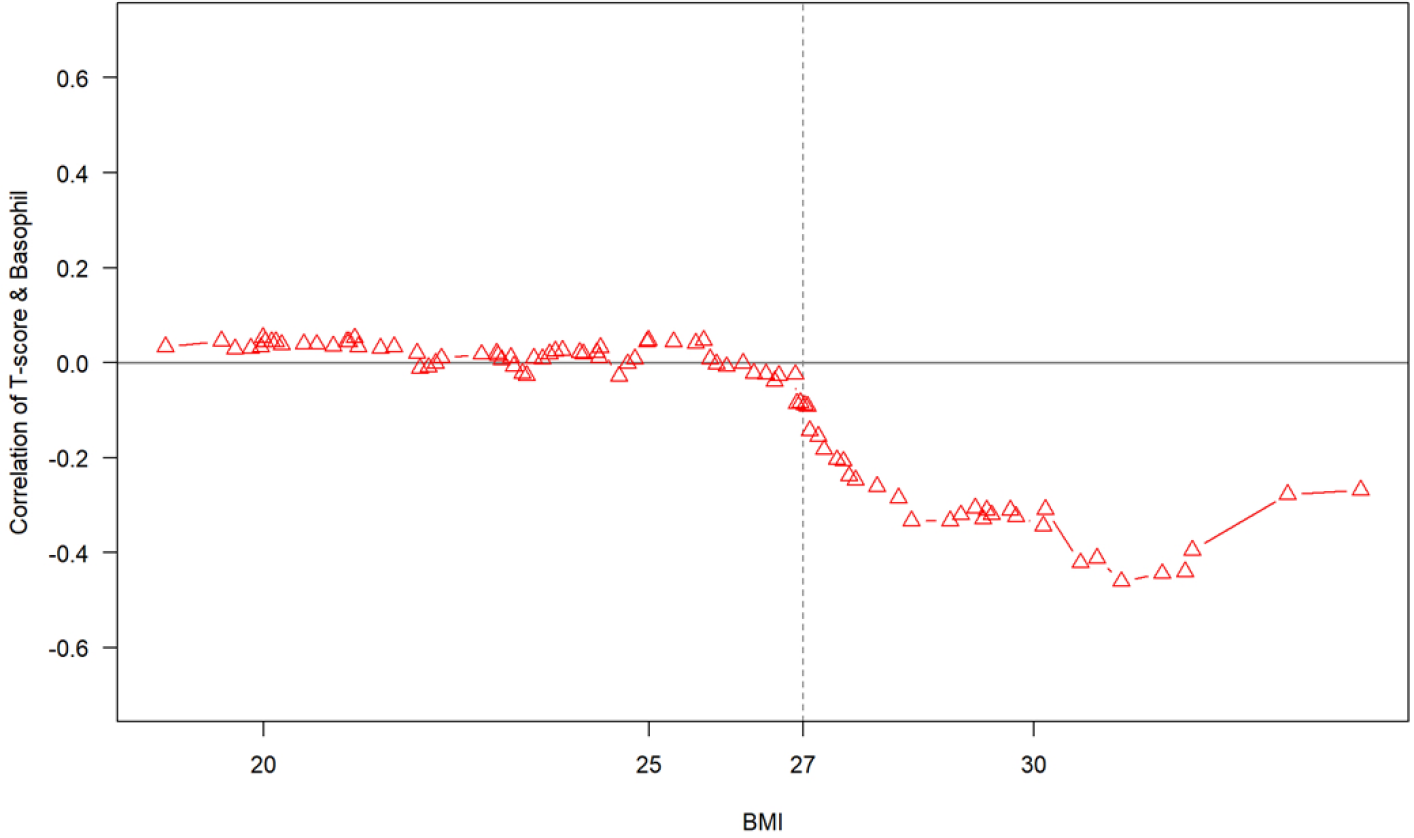
Scatterplots illustrating the correlation between lumbar spine T-score and basophil count across BMI-defined subgroups. A stepwise analysis across different BMI categories revealed that in individuals with BMI ≥ 27, T- score began to show a negative correlation with basophil count. This pattern was not evident in lower BMI groups. These findings suggest that the relationship between immune activity and bone mineral density may vary depending on body composition, highlighting a potential interaction between adiposity and inflammatory mechanisms in skeletal health.

Age was significantly and negatively associated with T-score (p = 2.0e−10), confirming its dominant influence on BMD reduction. In contrast, height and weight showed positive correlations with T-score (both p < 0.001), suggesting that greater body size may confer a protective effect on bone mass. Although BMI showed only a modest correlation with T-score (r = 0.3, p = 1.3e−5), a negative association between basophil count and BMD emerged in individuals with BMI ≥ 27. This finding suggests that in the context of elevated body weight, immunological factors such as basophil levels may contribute to bone loss, possibly through metabolic or inflammatory pathways. (**Figures 3**, **4**).

**Figure 5:**
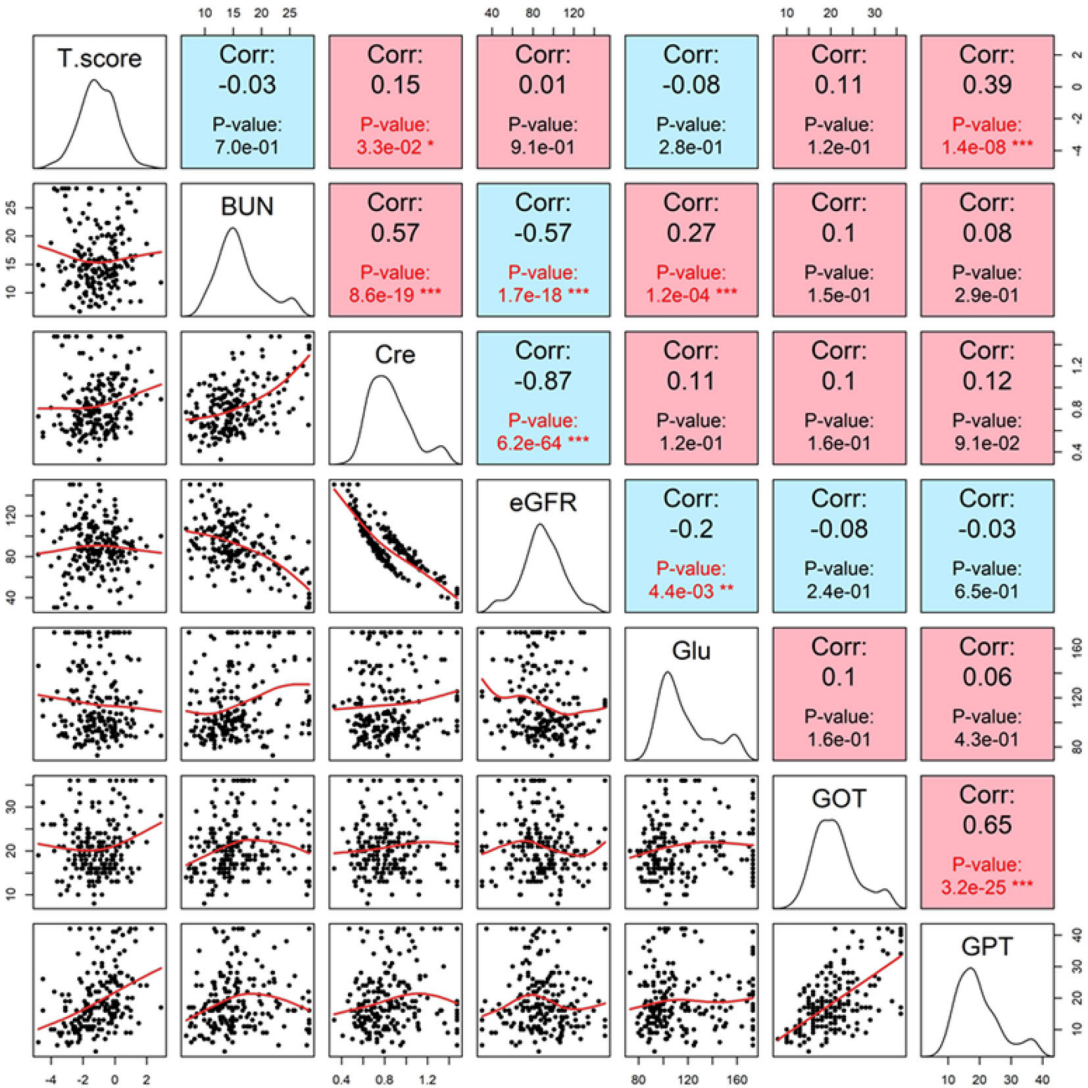
Pairwise correlation plots between T-score and key biochemical parameters including renal (creatinine, eGFR, BUN), hepatic (GOT, GPT), and glucose indices. GPT showed the strongest positive correlation with T-score (r = 0.39, p=1.4e-8), while creatinine was inversely correlated (r = 0.15, p =3.3e-2). These associations underscore the interplay between metabolic function and bone density in the studied population.

Among the biochemical markers, alanine aminotransferase (GPT) was significantly and positively associated with T-score in both univariate (r = 0.39, p = 1.4e−8). Serum creatinine demonstrated a weak positive correlation with T-score in univariate analysis (r = 0.15, p = 3.3e−02), suggesting a possible, yet inconclusive, link between renal function and BMD (**Figure 5**).

**Figure 6:**
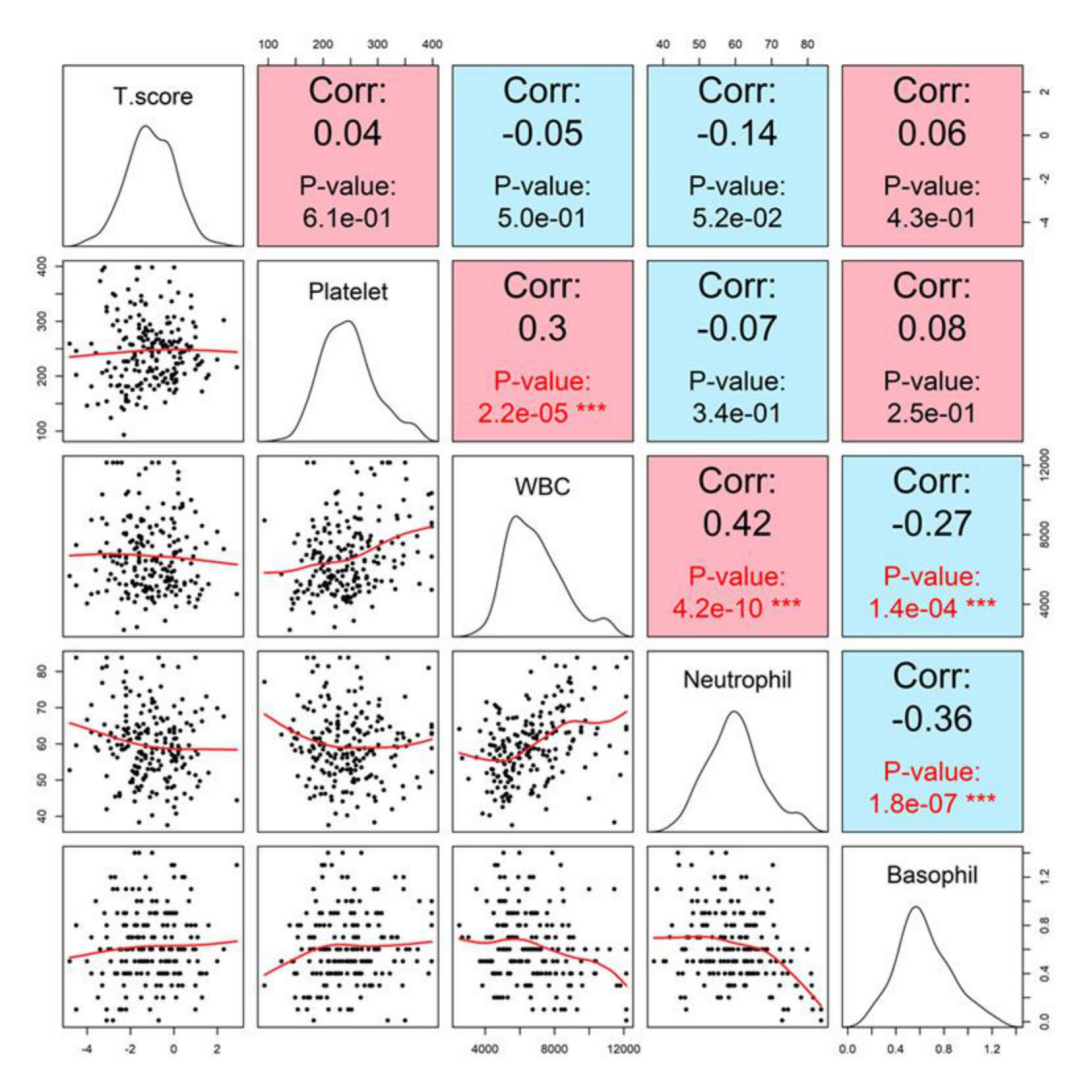
Correlation matrix with lumbar spine T-score. Pairwise scatterplots and Pearson correlation coefficients between T-score and hematologic markers, including white blood cell count (WBC), neutrophils, basophils, and platelets. None of the markers demonstrated statistically significant correlations with T-score. Specifically, WBC showed a very weak negative correlation (r = -0.05, p = 0.50), platelet showed a weak positive correlation (r = 0.04, p = 0.61), neutrophil showed a weak negative correlation (r = −0.14, p = 0.052), and basophil showed a weak positive correlation (r = 0.06, p = 0.43).

Basophil count demonstrated only a negligible correlation with T-score (r = 0.06, p = 0. 4261). Other hematologic indices, including white blood cell (WBC) and neutrophil counts, showed no significant correlation with T-score in either univariate or group-based analyses (**Figure 6**).

Using repeated stratified random sampling, multivariate linear regression incorporating age, body weight, WBC count, platelet count, basophil count, neutrophil count, creatinine, glucose, and GPT identified age (β = −1.91), body weight (β = 0.91), and GPT (β = 0.61) as the strongest independent predictors of T-score. Other variables showed weaker associations, including platelet (β = 0.1693), WBC count (β = −0.2855), neutrophil count (β = 0.3947), basophil count (β = 0.2405), creatinine (β = 0.3776), and glucose (β = 0.3933). The model intercept was −0.0884.

## Discussions

This study was conducted to evaluate the relationship between peripheral blood basophil count and bone mineral density (BMD) in an East Asian adult population (48). Previous studies have shown well established associations between osteoporosis and conventional risk factors such as age, sex, hormonal imbalance, and vitamin D deficiency (12–16, 18, 39). More recently, systemic inflammation has been recognized as an additional contributor to bone loss, with markers such as neutrophil-to-lymphocyte ratio (NLR) and C-reactive protein (CRP) attracting significant interest (19, 22–24, 33–38). Basophils, though rare among circulating granulocytes, have been shown to release histamine, leukotrienes, interleukin-4 (IL-4), and interleukin-13 (IL-13), which are hypothesized to influence osteoclastogenesis and bone resorption (56–58). Nonetheless, existing evidence regarding their role in bone metabolism remains limited and inconclusive (28, 59). In the present study, no statistically significant linear correlation was observed between basophil count and lumbar spine T-score in the overall cohort (Pearson’s r = 0.06, p = 0. 4261). This suggests that basophil count alone is unlikely to serve as a reliable biomarker for bone mineral loss in the general East Asian population. However, stratified analysis based on body mass index (BMI) revealed that among individuals with BMI ≥ 27, a weak inverse correlation emerged between basophil count and T-score, although it remained statistically non-significant. These findings suggest that the association between basophils and BMD may be modulated by host metabolic status, indicating a context-specific immunometabolic interaction. Obesity is associated with chronic low-grade inflammation, which alters the immune cell profile and cytokine environment, potentially affecting basophil function (60).

By contrast, significant associations were identified for liver and renal biomarkers. GPT (alanine aminotransferase) demonstrated a consistent and significant positive correlation to T-score (Pearson’s r = 0.39, p =1.4e−8), suggesting that liver function may serve as a surrogate marker of systemic metabolic status affecting bone health. Creatinine also showed a positive association to T-score (r = 0.15, p = 3.3e−2). These results underscore the potential role of hepatorenal function in modulating bone density, possibly through pathways involving energy metabolism, protein turnover, or systemic inflammation.

Our findings align with prior reports indicating that chronic inflammation may play a role in osteoporosis. However, most existing studies have focused on neutrophils and lymphocytes, with minimal attention given to basophils. This study extends the literature by identifying a potential link between elevated basophil count and reduced BMD, particularly within an East Asian context, where osteoporosis prevalence continues to rise. While the statistical strength of the association remains limited, the potential clinical applicability of basophil count as a supplementary marker for bone health risk stratification warrants further validation.”

**Figure 7:**
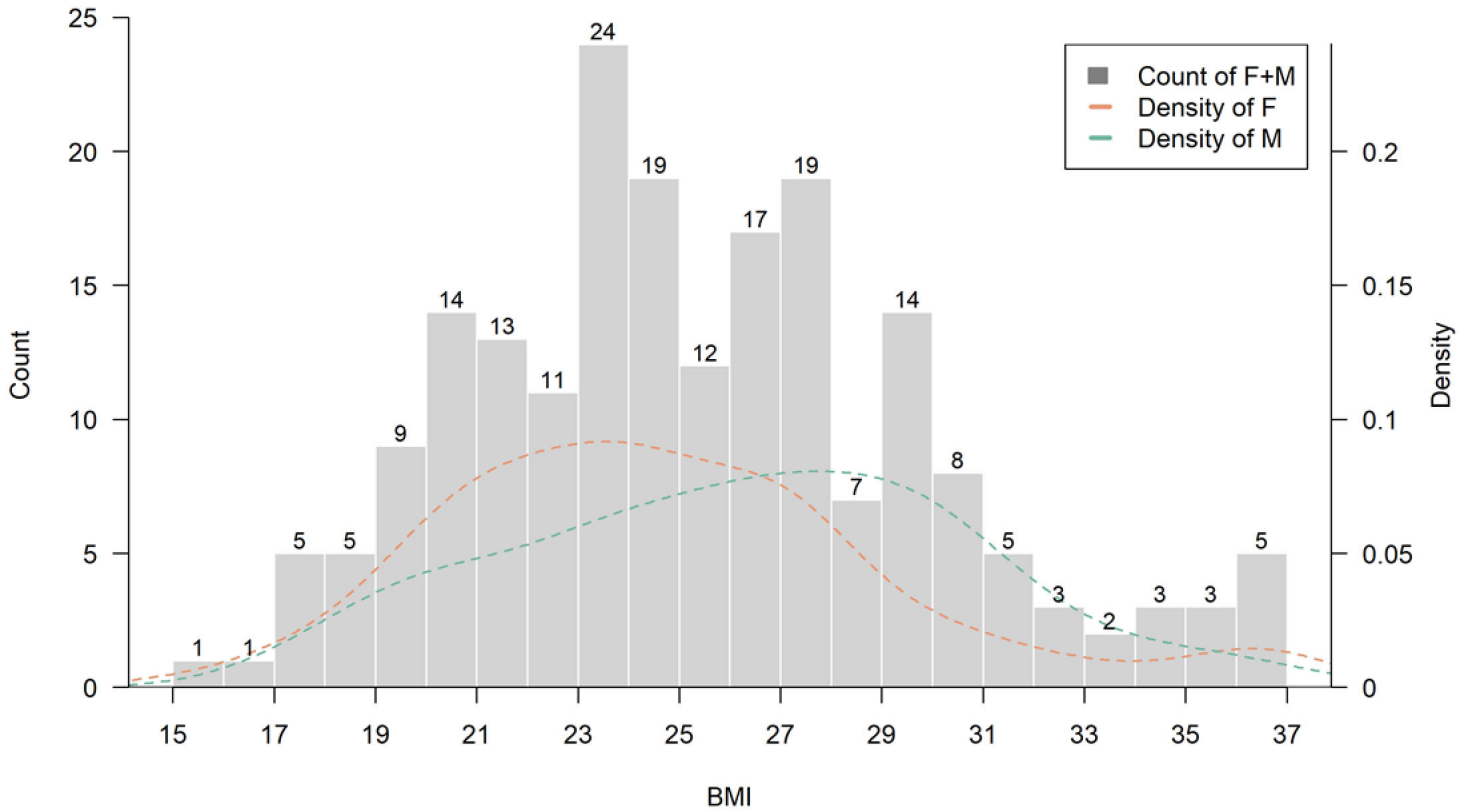
Distribution of body mass index (BMI) among study participants (N = 200). This histogram illustrates the distribution of BMI values within the study cohort. The overall pattern approximates a normal distribution, with a calculated mean BMI of 25.28 ± 4.37 kg/m^2^ and a median of 24.97 kg/m^2^. The highest concentration was observed in the 23-27 kg/m^2^ range. Density curves were plotted separately for male and female participants.

From a clinical standpoint, integrating basophil count into routine risk screening protocols could offer an inexpensive adjunct to existing osteoporosis risk models. Its availability in standard blood panels enhances its potential for widespread implementation in both hospital and community-based settings. Future health policy frameworks may consider incorporating basophil count into osteoporosis screening guidelines, especially in aging populations with limited access to advanced diagnostics. Additionally, BMI was positively associated with T-score (r =0.30, p = 1.3e−5). Interestingly, in participants with a BMI greater than 27, basophil counts began to show a negative correlation with T-scores, suggesting a potential inflammatory mechanism that may offset the protective effects of increased body mass on bone integrity (**Figures 3**, **4**). Approximately 69 participants fell into this higher BMI category, which may reflect dietary and genetic patterns characteristic of East Asian or Austronesian-admixed populations. While anthropologic interpretations should be approached with caution, this finding highlights the necessity of considering population-specific body composition trends in musculoskeletal research and clinical evaluation (**Figure 7**).

Taken together, these results contribute to the growing field of osteoimmunology. While inflammatory indices such as NLR and CRP have shown reproducible associations with osteoporosis across cohorts in postmenopausal women (19, 22–24, 28–33), the contribution of basophils appears limited. Despite their known immunomodulatory functions and accessibility via routine blood tests, basophil counts did not independently predict BMD in this study. When compared to well-established predictors—such as age, sex, GPT, and creatinine—the quantitative impact of basophils was minimal.

These findings, although preliminary, support the exploration of hematologic biomarkers as potential screening alternatives or adjunct diagnostic markers for BMD loss. Because basophil count, alanine aminotransferase (GPT), and creatinine are easily and routinely measured during standard health examinations, incorporation of these markers into multivariate prediction tools or artificial intelligence-based risk models could improve early detection and personalized intervention for osteoporosis (46, 52–54). Recent studies have explored the integration of blood-based biomarkers into artificial intelligence frameworks to enhance osteoporosis risk prediction accuracy and reduce reliance on imaging in low-resource settings (61–63). Combining these biomarkers with imaging modalities such as DEXA or emerging portable bone scanners may also enable hybrid diagnostic strategies tailored to the needs of specific ethnic groups. Nevertheless, the findings support the continued exploration of hematologic and metabolic profiles in BMD assessment. The weak but detectable associations observed in specific subgroups suggest that basophils may still serve a complementary role within broader immunoinflammatory indices. Further prospective studies incorporating dynamic biomarker monitoring, vitamin D status, parathyroid hormone (PTH) levels, and lifestyle factors will be crucial for elucidating the precise mechanisms underlying immune-mediated bone remodeling in diverse populations.

## Conclusions

This study provides updated evidence on the relationship between clinical and biochemical parameters and bone mineral density (BMD) in an East Asian adult population. As expected, age and female sex remained the most robust predictors of lower lumbar spine T-scores. Among biochemical indices, alanine aminotransferase (GPT) emerged as a consistent and independent positive predictor of BMD.

Although basophil count has been previously hypothesized to influence bone metabolism through immunomodulatory mediators such as histamine, IL-4, and IL-13, the present study found only a negligible correlation with T-scores (r = 0.06). Subgroup trends suggesting a possible inverse association in individuals with elevated BMI warrant further investigation but do not support basophil count as a reliable standalone biomarker for osteoporosis risk stratification.

By integrating hematologic and biochemical markers into a multivariate framework, this study contributes to the evolving field of osteoimmunology. The findings highlight GPT as a potentially useful surrogate marker for BMD and underscores the need for future research to clarify the mechanistic links between systemic metabolic status and skeletal health across diverse populations.

Furthermore, the potential for integrating hematologic markers into low-cost, scalable diagnostic frameworks should be further investigated, particularly in aging populations with limited access to imaging-based screening. Our findings suggest that routinely tested biochemical parameters may hold value for osteoporosis risk stratification, particularly in settings lacking access to DXA.

## Limitations

Several limitations of this study should be acknowledged. First, although basophil count demonstrated a negligible correlation with BMD (r = 0.06) in univariate analysis, a negative association began to emerge in the subgroup of individuals with BMI ≥ 27. This suggests that the relationship may be context-dependent, potentially influenced by metabolic or inflammatory changes in overweight individuals, rather than representing a universal predictive factor.

Second, the sample size (n = 200) was relatively modest and derived from a single regional medical center, potentially limiting statistical power and generalizability. Third, several important determinants of bone health—including serum vitamin D, PTH levels, calcium-phosphate homeostasis, corticosteroid exposure, and physical activity—were not available in the dataset and could not be accounted for in the analysis.

Menopausal status was not available in our retrospective dataset. While prior studies, such as Leeyaphan et al. (28), focused exclusively on postmenopausal women, our analysis included a broader population. This limits direct comparability, but also allows for hypothesis generation across age groups. Future prospective studies should aim to stratify by menopausal status to clarify potential modifying effects. Although sex and age are potential confounding factors, subgroup analyses by gender and age stratification were limited by sample size. Future studies with prospective designs and menopausal status records are warranted. In addition, systemic inflammation markers such as the neutrophil-to-lymphocyte ratio (NLR) and systemic immune-inflammation index (SII) were not included in the analysis due to the absence of complete lymphocyte data in the retrospective cohort. This limited our ability to evaluate broader inflammatory patterns or validate the observed neutrophil–BMD relationship within established frameworks. Future studies with complete differential counts may help determine whether basophil-associated trends persist after adjusting for composite inflammatory indices.

Furthermore, the study cohort consisted exclusively of East Asian individuals from a single geographic region, which may limit the extrapolation of findings to other ethnic groups or populations with differing genetic and environmental risk factors. Despite these limitations, the study offers novel insights into the metabolic and immunologic correlates of BMD and underscores the importance of region-specific data in refining osteoporosis risk stratification and prevention strategies.

## Data Availability

The original contributions presented in the study are included in the article/supplementary material. Further inquiries can be directed to the corresponding authors.
